# Supplementary material for: Design and Synthesis of New Sulfonic Acid Functionalized Ionic Liquids as Catalysts for Esterification of Fatty Acids with Bioethanol
Source: Molecules. 2023 Jul 5;28(13):5231. doi: 10.3390/molecules28135231 (PMC10343253; doi:10.3390/molecules28135231)
Supplement: Supplementary file 1 [file molecules-28-05231-s001.zip › molecules-2431935-supplementary.pdf]

Supplementary Material for

Design and Synthesis of New Sulfonic Acid Functionalized Ionic Liquids as Catalysts for Esterification of Fatty Acids with Bioethanol

Nguyen Thi T. H., Koutecká J., Kaule P., Vrtoch L., Šícha V., Čermák J*.

Jan Evangelista Purkyně University in Ústí nad Labem, Faculty of Science, Pasteurova 15, 400 96 Ústí nad Labem, Czech Republic, e-mail: cermak@icpf.cas.cz

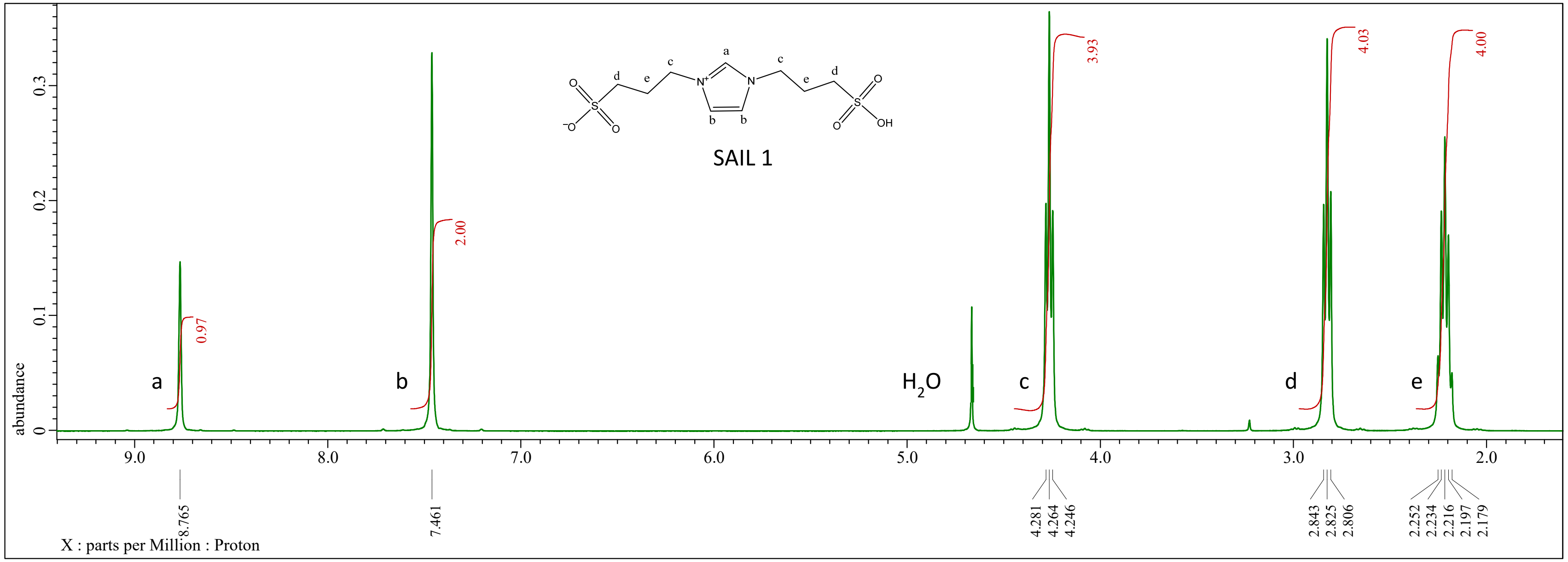

Figure S1. ^{1}H NMR spectra of SAIL **1.** Lower case letters in this and subsequent spectra show the assignment.

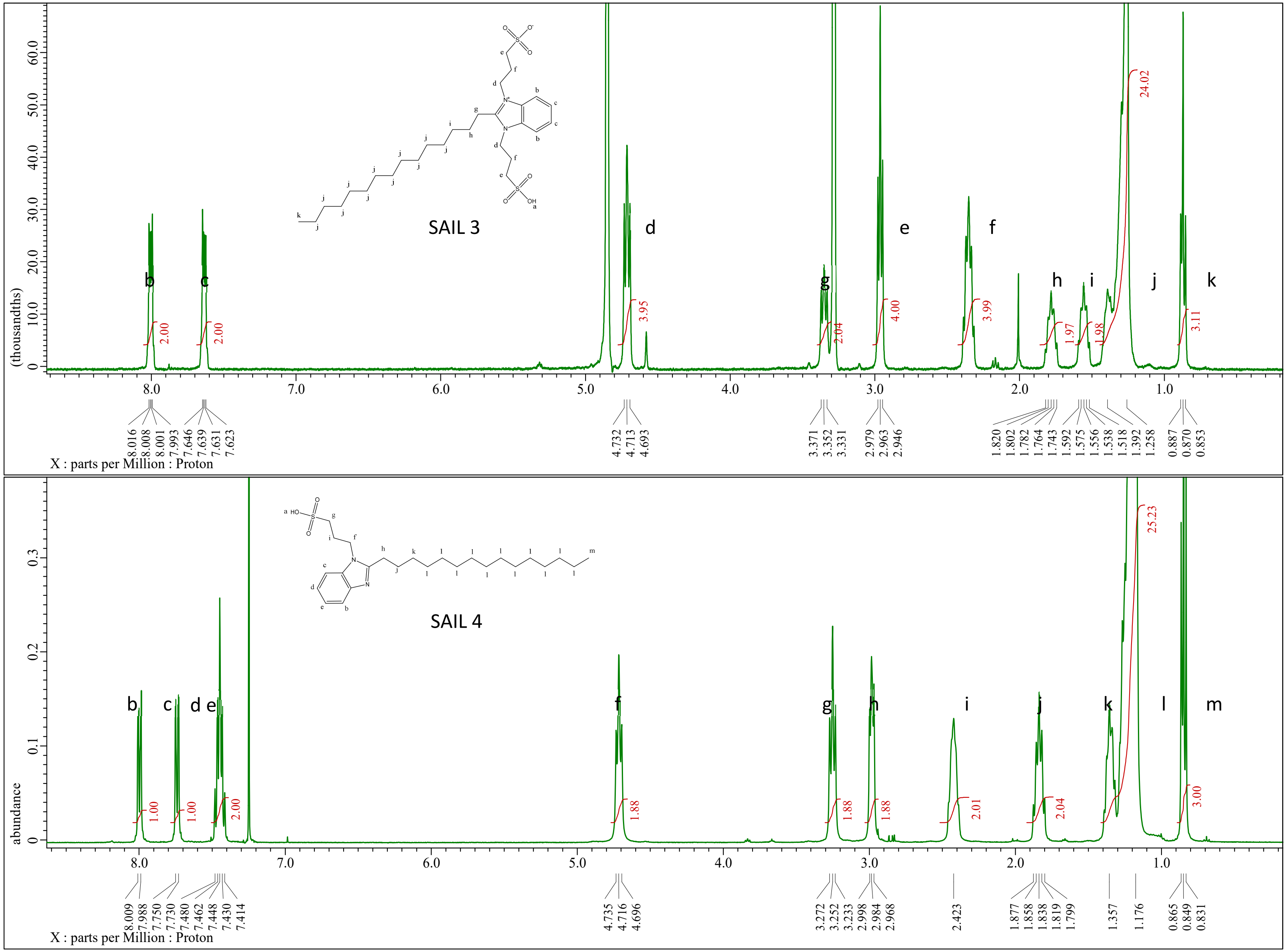

Figure S2. ^{1}H NMR spectra of SAIL **3, 4**

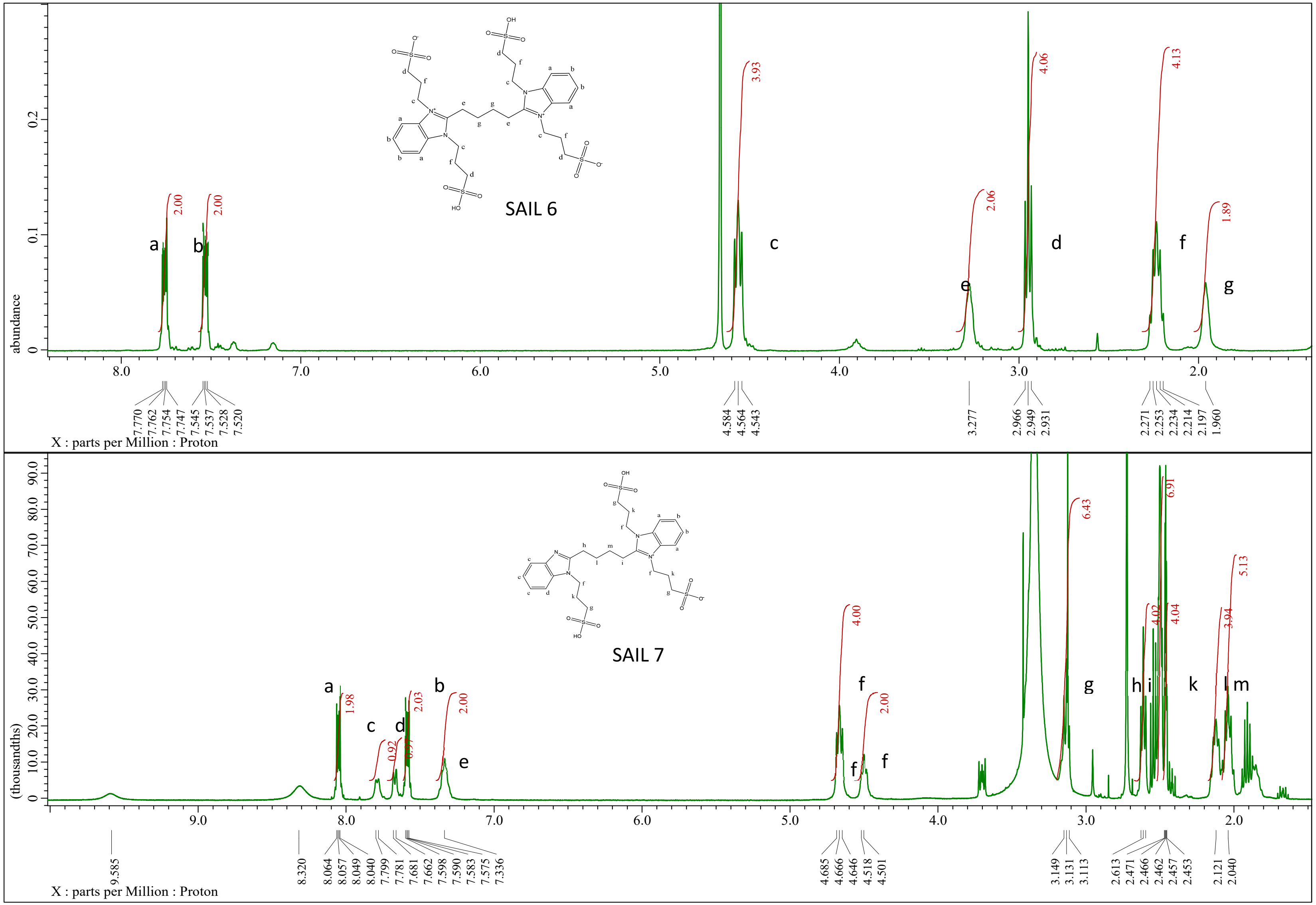

Figure S3. ^{1}H NMR spectra of SAIL **6, 7**

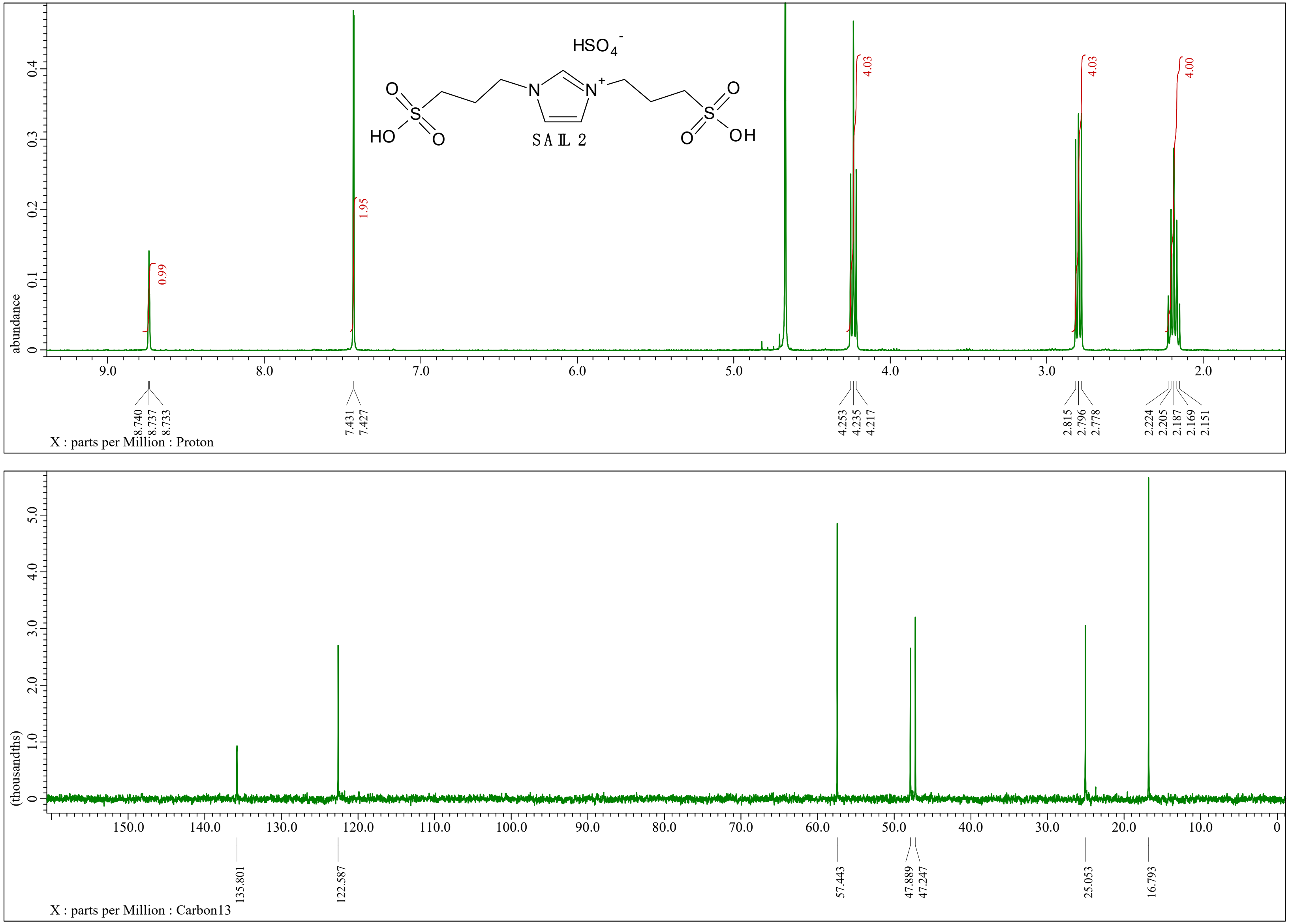

Figure S4. ^{1}H, ^{13}C-NMR spectra of SAIL **2**

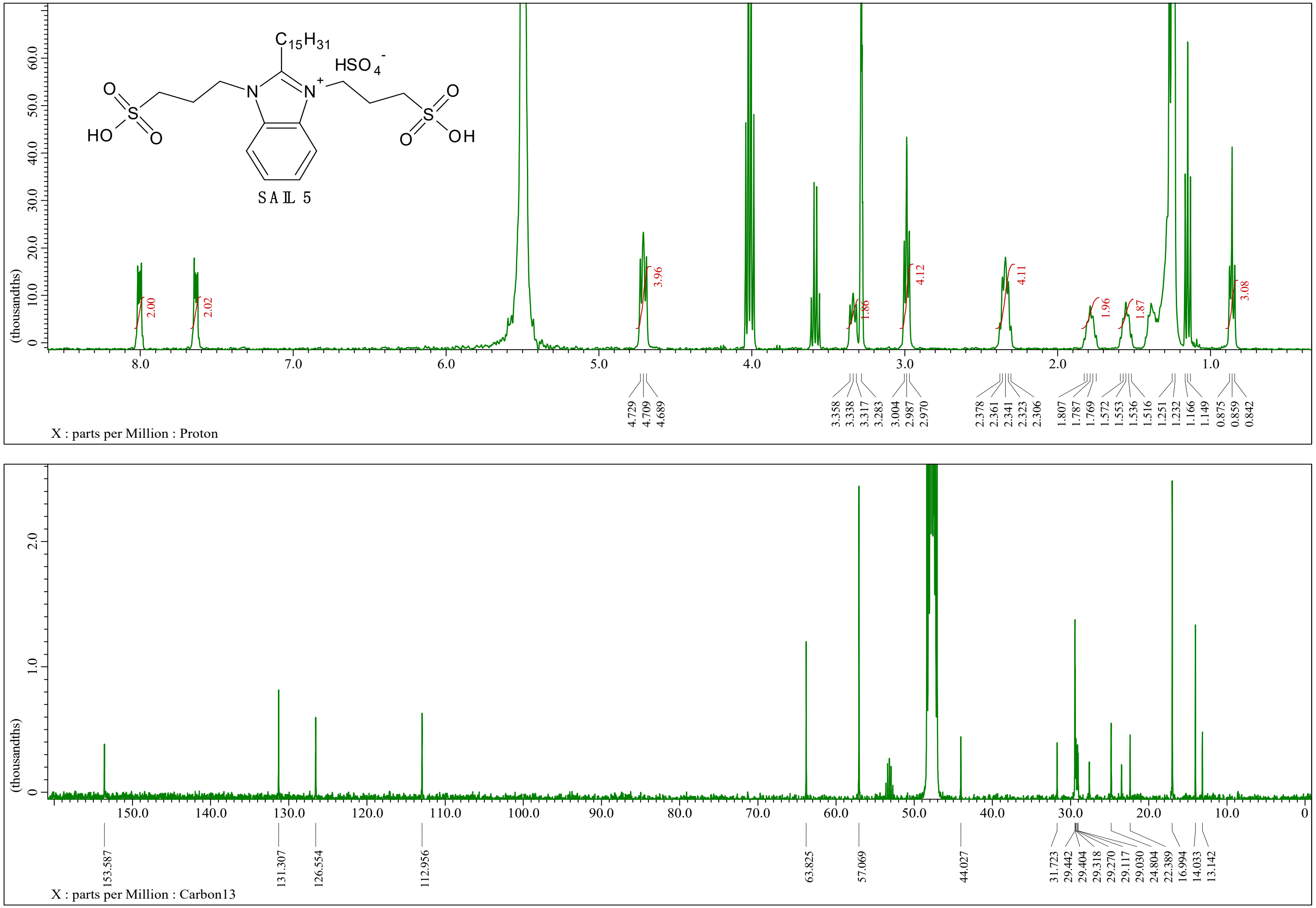

Figure S5. ^{1}H, ^{13}C-NMR spectra of SAIL **5**

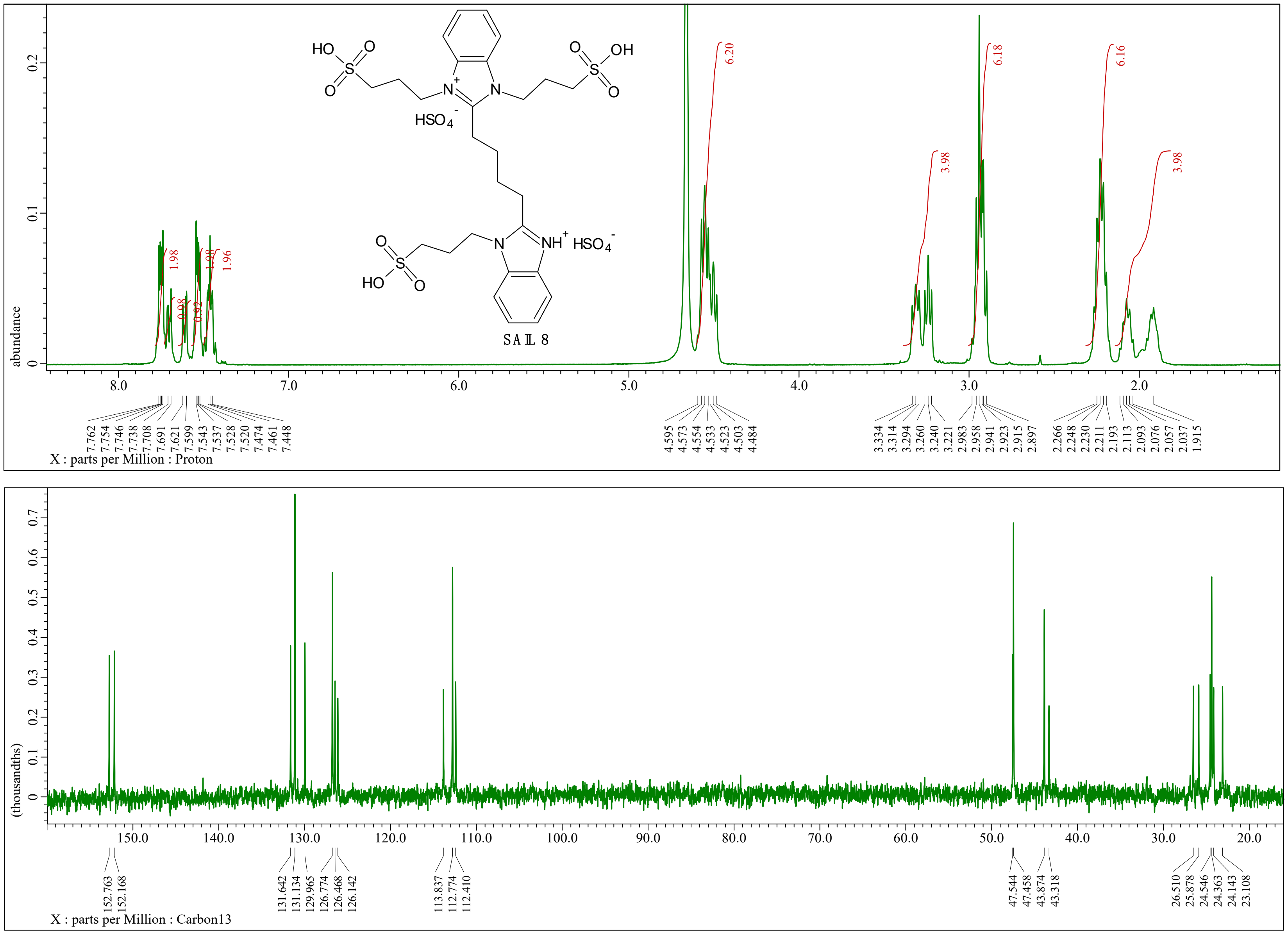

Figure S6. ^{1}H, ^{13}C-NMR spectra of SAIL **8**

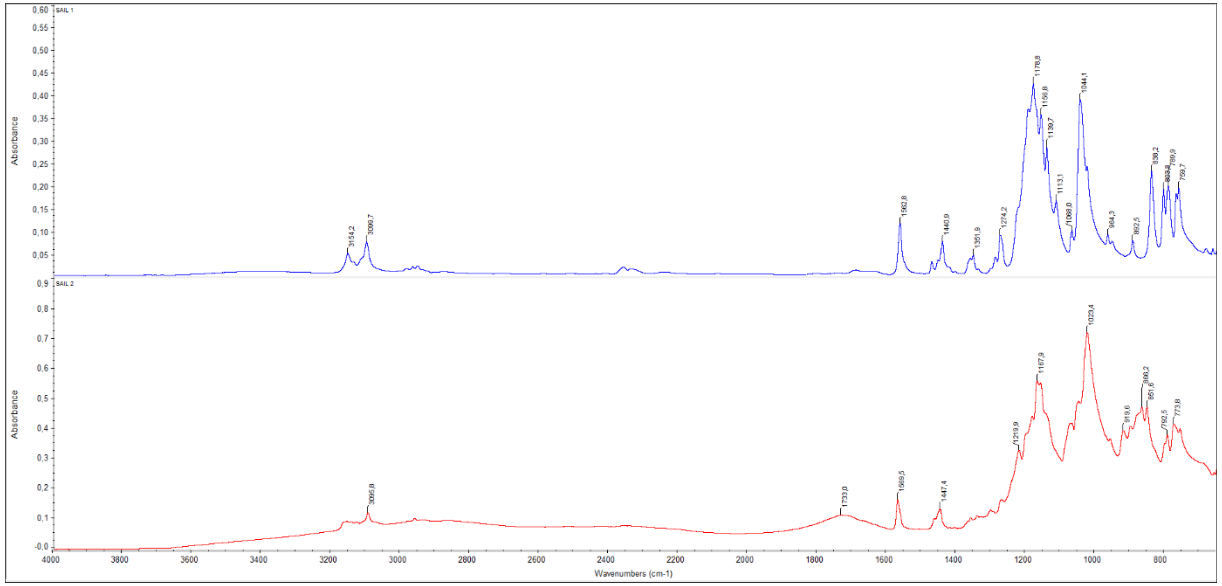

Figure S7. IR spektra of SAIL **1, 2**

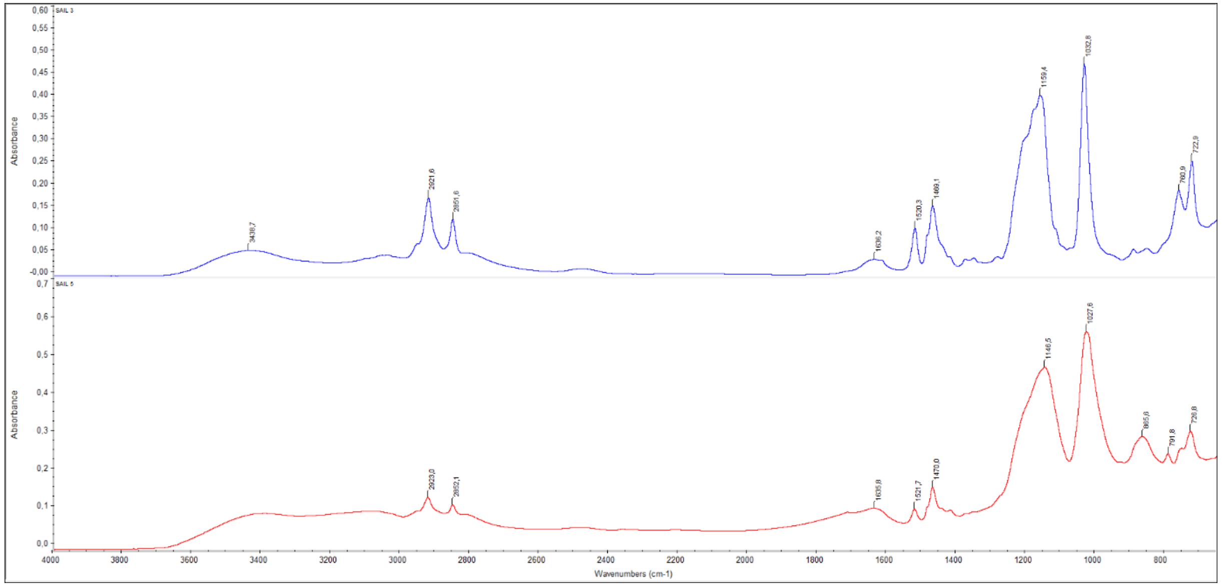

Figure S8. IR spektra of SAIL **3, 5**

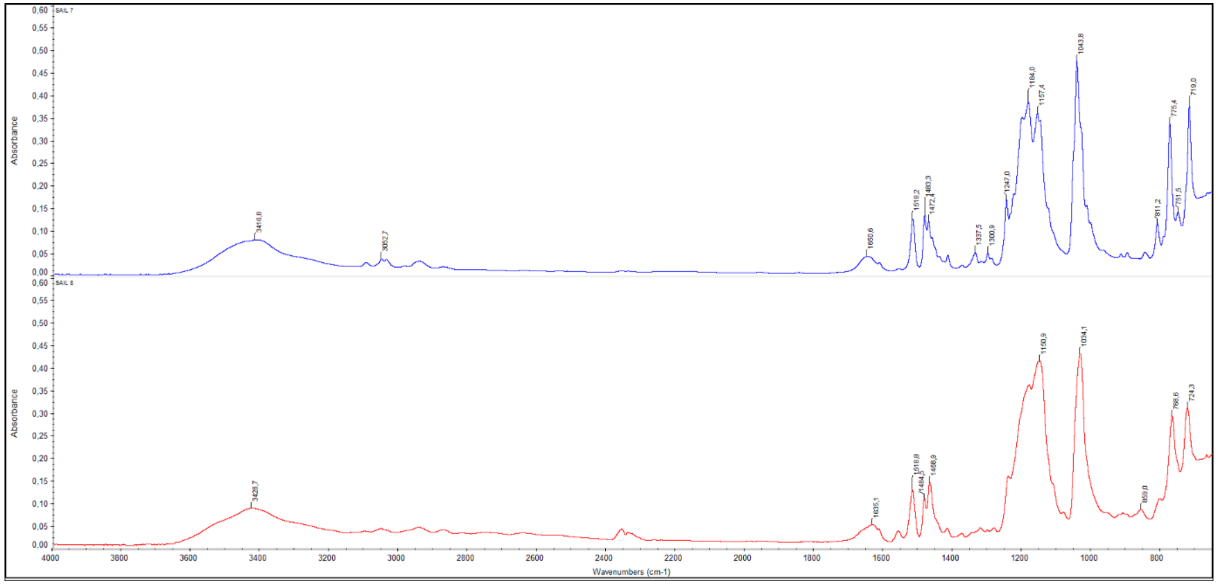

Figure S9. IR spektra of SAIL **7, 8**

As can be seen from Fig. 2(SAIL1, 2), for the structure **1**, **2** the bands at 1179 resp. 1168 cm^{-1} were assigned to the O=S=O stretching vibrations, the 1044 resp. 1023 cm^{-1} band was assigned to the C-S-O stretching vibrations. For the structure **3**, **5** the bands at 1170 resp. 1168 cm^{-1} were assigned to the O=S=O stretching vibrations, the 1030 resp. 1023 cm^{-1} band was assigned to the C-S-O stretching vibrations. For the structure **7**, **8** the bands at 1157 resp. 1151 cm^{-1} were assigned to the O=S=O stretching vibrations, the 1044 resp. 1034 cm^{-1} band was assigned to the C-S-O stretching vibrations. These results showed the existence of sulfonic acid group.

The band at 3154, 3100 were assigned to the unsaturated C-H and 1563, 1441 to ring stretching vibration of imidazole. The wide peak at bands at 3430, 3100 was assigned to the unsaturated C-H and 1636, 1520, 1470 to ring stretching vibration of benzimidazole.

The band at 2950 - 2850 were assigned to the saturated alkyl chain saturated C-H stretching vibration. Strong bands at 2922, 2852 in Fig 2(SAIL3, 5) were assigned to the C-H stretching vibration of long saturated alkyl chain $C_{15}H_{31}$.

MASS SPECTROMETRY DATA

Table S1. An overview of LCQ Fleet IonTrap ESI mass spectrometry characterized SAIL´s samples with comparison of obtained and calculated masses of molecular cations, and summary formulae.

SAIL	$[M+H]^+$ exp.	$[M+H]^+$ calc.	Summary Formula
1	313.14	313.05	$C_9H_{17}N_2O_6S_2$
2	313.08	313.05	$C_9H_{17}N_2O_6S_2$
3	573.56	573.40	$C_{27}H_{61}N_2O_6S_2$
4	451.32	451.30	$C_{25}H_{43}N_2O_3S_1$
5	573.44	573.30	$C_{28}H_{49}N_2O_6S_2$
6	799.26	799.14	$C_{30}H_{43}N_4O_{12}S_4$
7	657.12	657.17	$C_{27}H_{37}N_4O_9S_3$
8	657.20	657.17	$C_{27}H_{37}N_4O_9S_3$

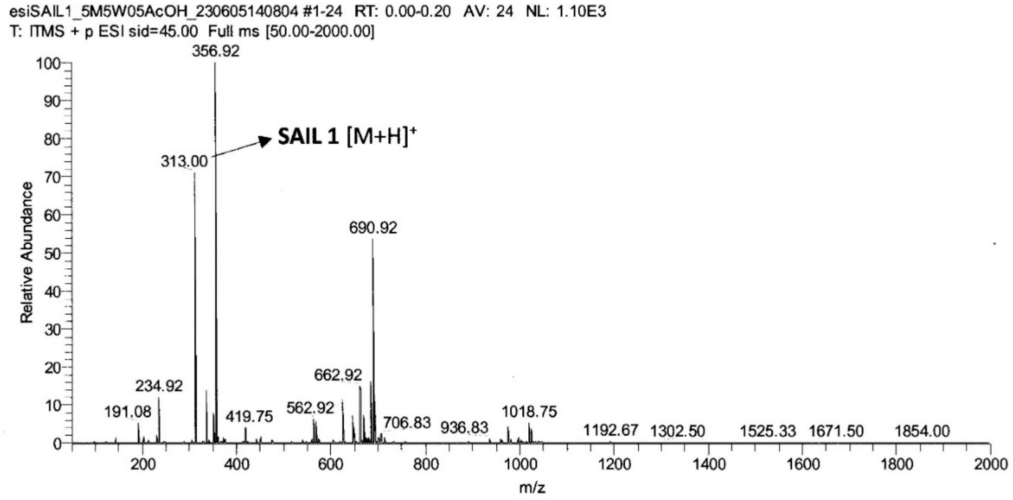

Figure S10. The full scan ESI MS spectrum of **SAIL 1**.

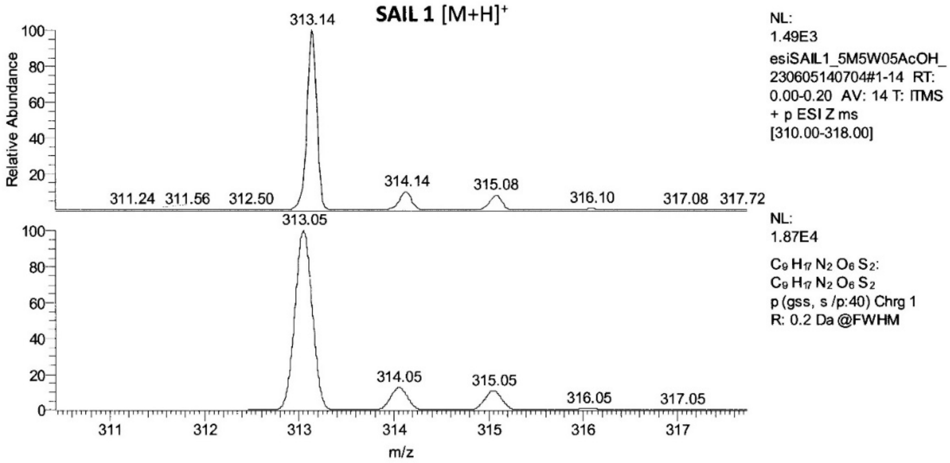

Figure S11. The zoom scan ESI MS spectrum of **SAIL 1**.

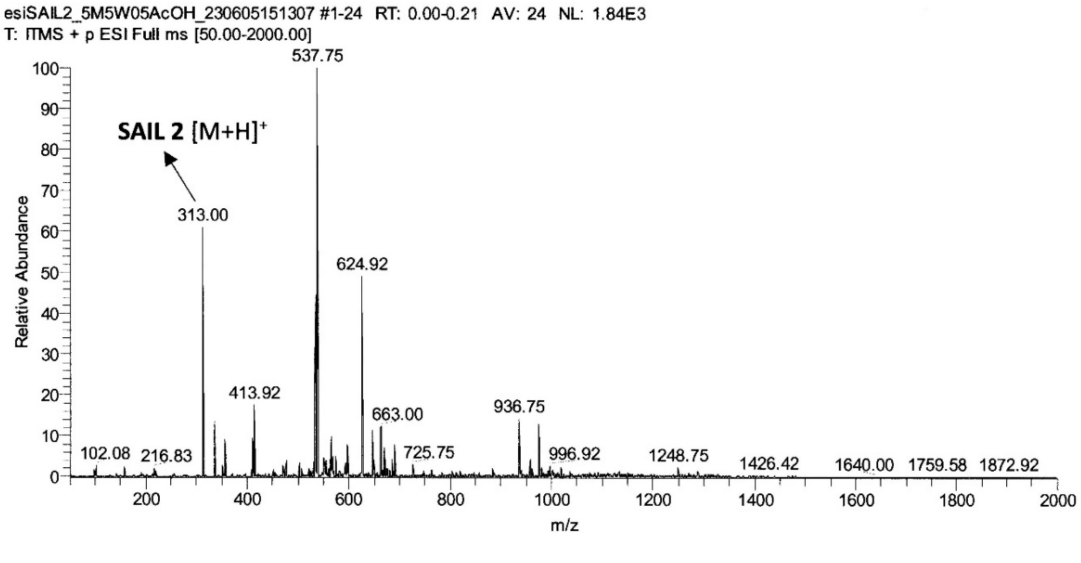

Figure S12. The full scan ESI MS spectrum of **SAIL 2**.

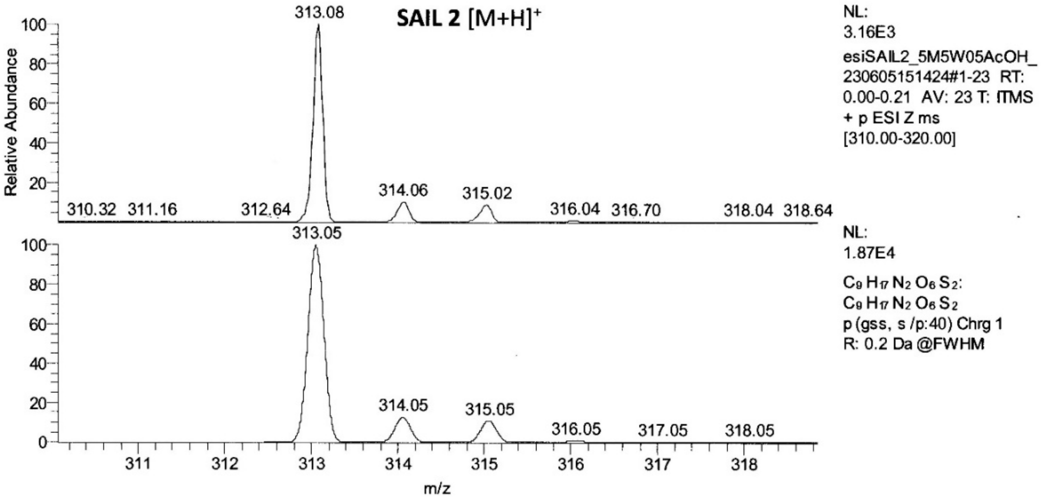

Figure S13. The zoom scan ESI MS spectrum of **SAIL 2**.

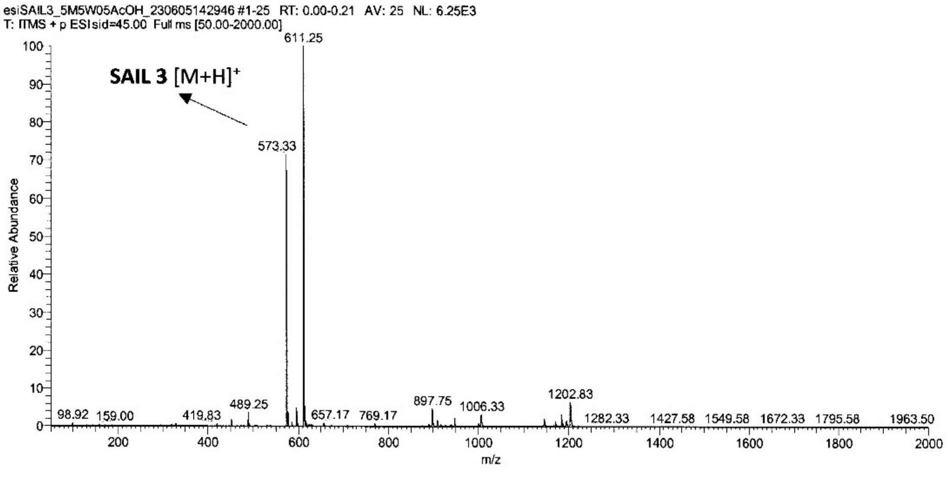

Figure S14. The full scan ESI MS spectrum of **SAIL 3**.

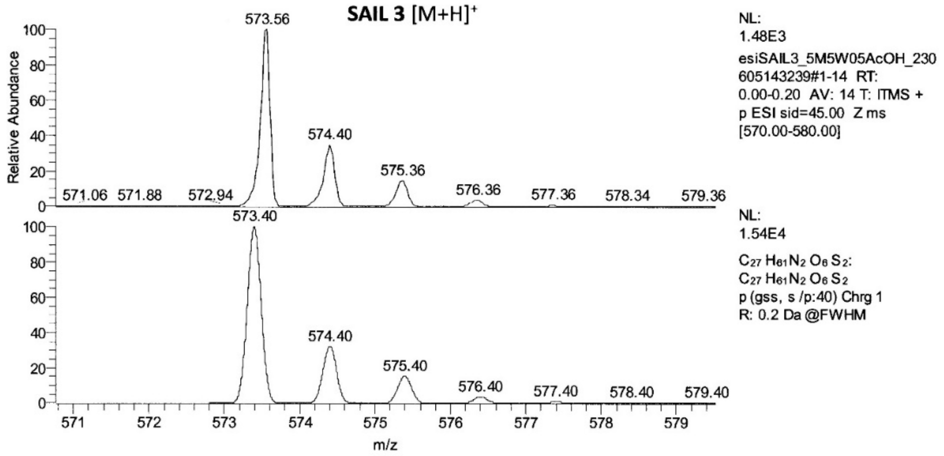

Figure S15. The zoom scan ESI MS spectrum of **SAIL 3**.

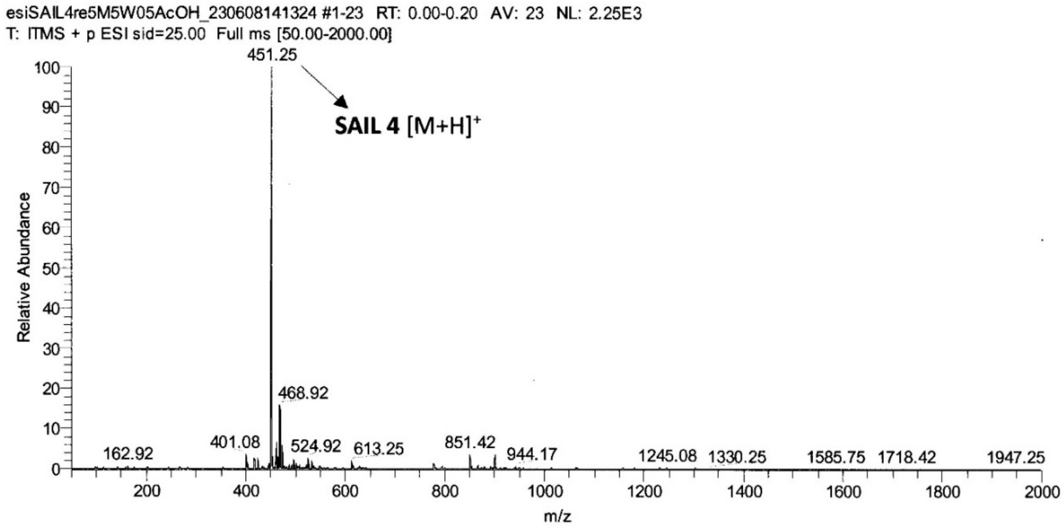

Figure S16. The full scan ESI MS spectrum of **SAIL 4**.

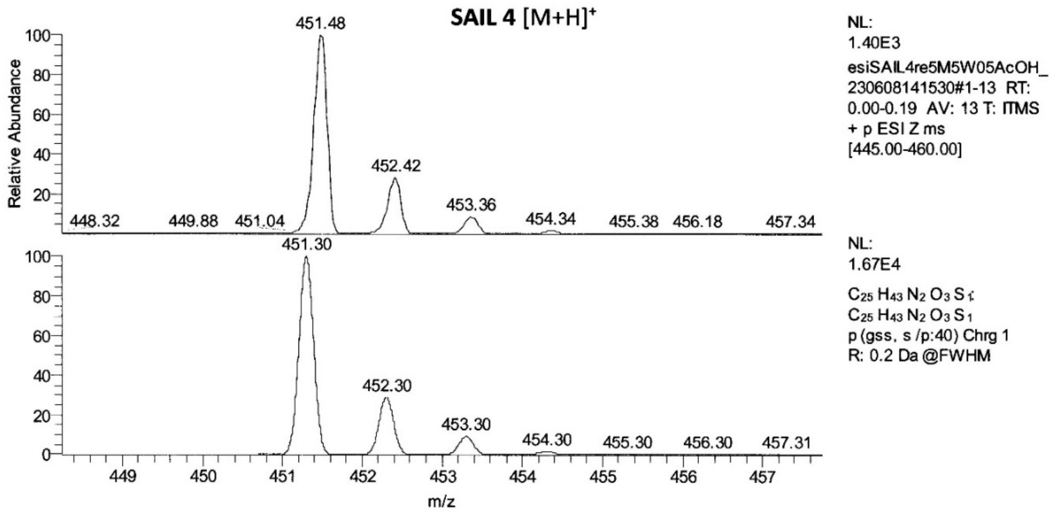

Figure S17. The zoom scan ESI MS spectrum of **SAIL 4**.

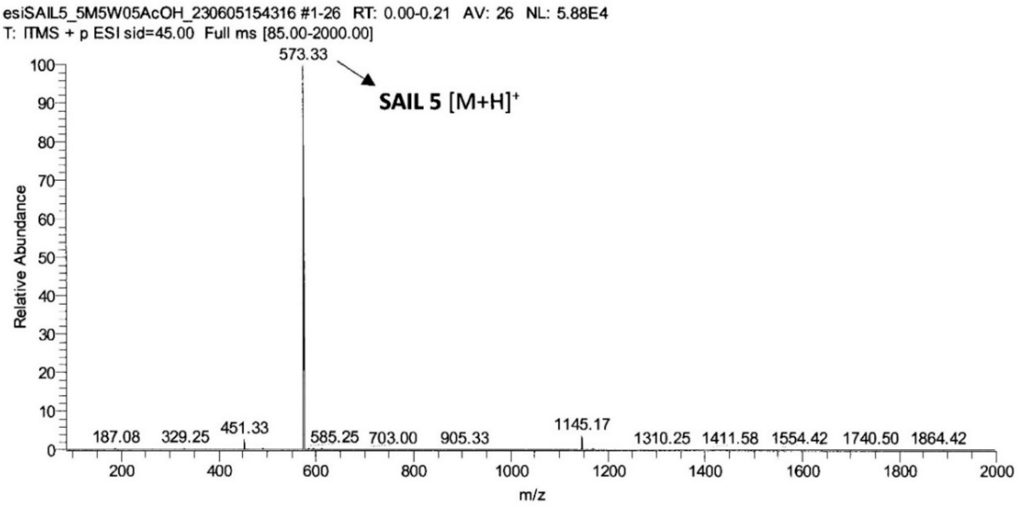

Figure S18. The full scan ESI MS spectrum of **SAIL 5**.

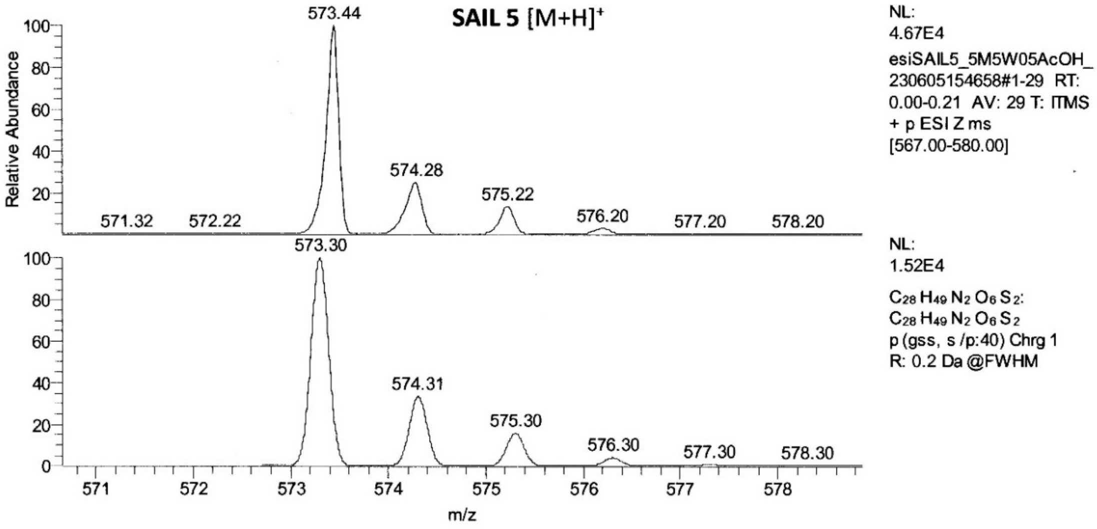

Figure S19. The zoom scan ESI MS spectrum of **SAIL 5**.

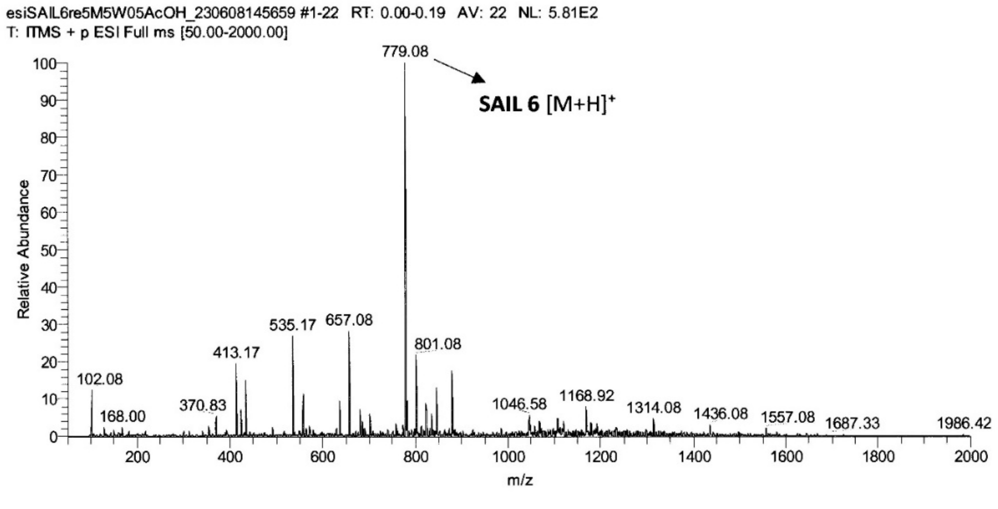

Figure S20. The full scan ESI MS spectrum of **SAIL 6**.

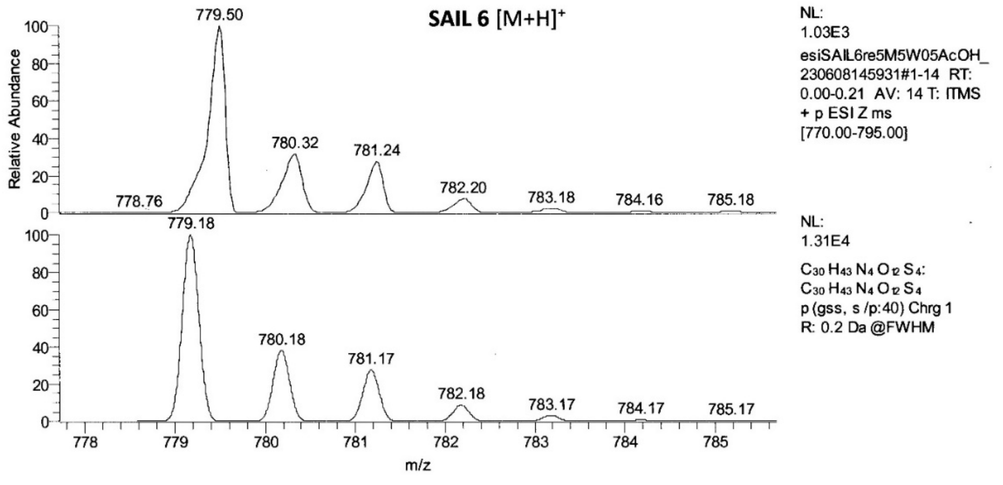

Figure S21. The zoom scan ESI MS spectrum of **SAIL 6**.

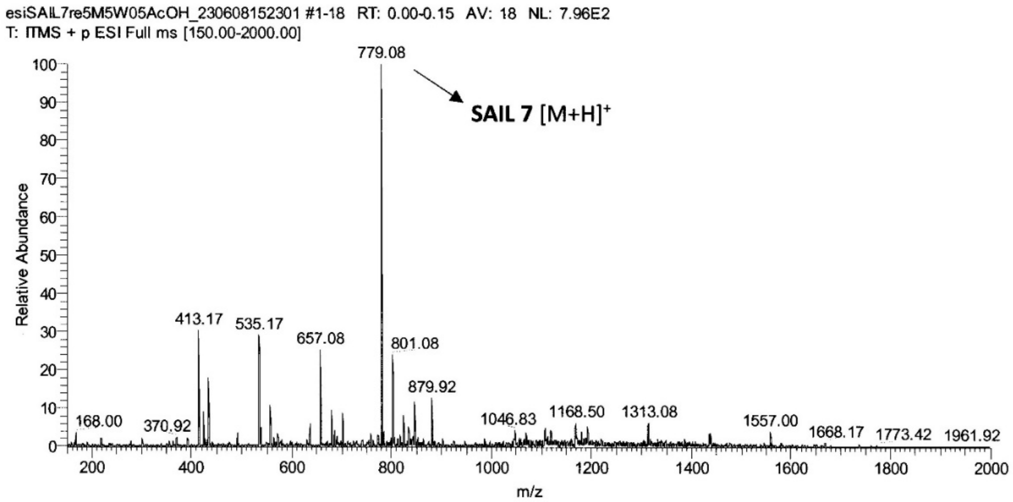

Figure S22. The full scan ESI MS spectrum of **SAIL 7**.

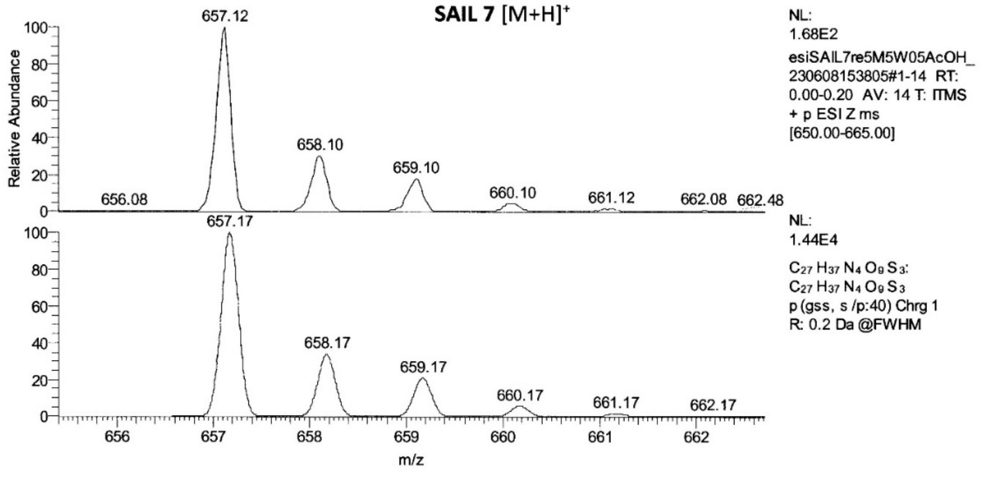

Figure S23. The zoom scan ESI MS spectrum of **SAIL 7**.

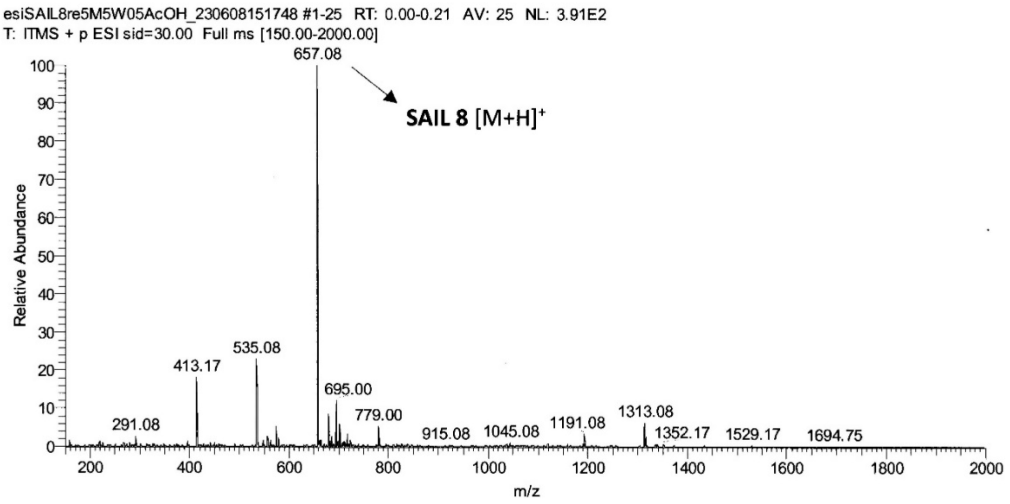

Figure S24. The full scan ESI MS spectrum of **SAIL 8**.

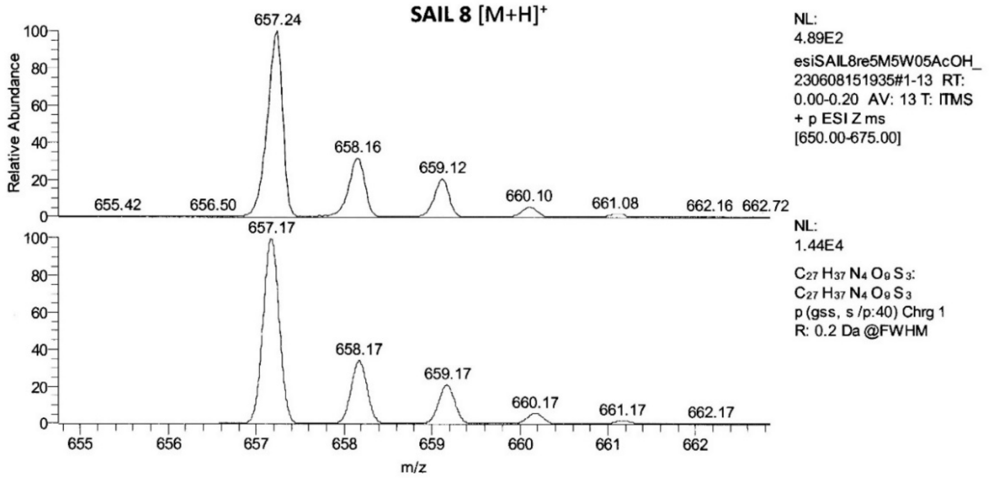

Figure S25. The zoom scan ESI MS spectrum of **SAIL 8**.
